# Chinese herbal medicine si-miao-san decoction for acute gouty arthritis: A protocol for systematic review and meta-analysis of randomized controlled trials

**DOI:** 10.1097/MD.0000000000021510

**Published:** 2020-08-07

**Authors:** Heting Wang, Hua Duan, Shiyin Chen, Yong Luo, Yuan Zhang, Qingsong Liu, Xiyu Zhang

**Affiliations:** a Hospital of Chengdu University of Traditional Chinese Medicine; b Sichuan Academy of Medical Sciences & Sichuan Provincial People's Hospital, Chengdu, Sichuan Province, P.R. China.

**Keywords:** acute gouty arthritis, protocol, si-miao-san decoction, systematic review

## Abstract

**Background:**

The prevalence of gout is increasing worldwide, and the symptoms of acute arthritis appearing in gout patients seriously affect the quality of life. The pain and functional limitation caused by acute gouty arthritis (AGA) bring great pain to patients. At present, mainstream drugs have problems such as poor efficacy and side effects. Traditional Chinese medicine has extensive clinical experience in the prevention and treatment of gout, and it also shows clear advantages in the treatment of AGA. Clinical studies have confirmed that si-miao-san decoction (SMSD), a traditional Chinese medicine decoction, can improve the clinical symptoms and signs of AGA patients. Therefore, we will conduct a systematic review to clarify the effectiveness and safety of SMSD for AGA.

**Methods:**

We will search different database from the built-in to October 2020. The electronic database includes PubMed, Embase, Cochrane Library, Web of Science, CNKI, WanFang, VIP, and CBM. At the same time, we will also search for clinical registration tests and gray literatures. This study only screened clinical randomized controlled trials (RCT) for SMSD for AGA. The 2 researchers independently conducted literature selection, data extraction, and quality assessment. Dichotomous data are represented by relative risk (RR), continuous data are represented by mean difference (MD) or standard mean deviation (SMD), and the final data are fixed effect model (FEM) or random effect model (REM), depending on whether it exists heterogeneity. The main outcomes are clinical efficacy, including pain score, joint function, and degree of swelling. The secondary outcomes include: blood uric acid (BUA), C-reactive protein (CRP), and erythrocyte sedimentation rate (ESR). Finally, a meta-analysis was conducted through Review Manager software version 5.3.

**Results:**

This study will conduct a comprehensive analysis based on the currently released Si-Miao-San data for the treatment of AGA and provide high-quality evidence of clinical efficacy and safety.

**Conclusion:**

This systematic review aims to provide new options for SMSD treatment of AGA in terms of its efficacy and safety.

**Ethics and dissemination:**

The review is based solely on a secondary study of published literatures and does not require ethics committee approval. Its conclusion will be disseminated in conference papers, magazines, or peer-reviewed journals.

**INPLASY registration number:**

INPLASY202040163.

## 1 Introduction

Gout is a metabolic disease characterized by hyperuricemia and monosodium urate (MSU) crystals deposited in joints and soft tissues. The main symptom of gout is acute inflammation of the joints. Acute gout is characterized by a sudden monoarthritis of rapid onset, with intense pain, mostly affecting the big toe (50% of initial attacks), the foot, ankle, midtarsal, knee, wrist, finger, and elbow.^[1]^ The incidence of gout has increased globally, and its incidence is largely consistent with as the prevalence of hyperuricemia increases.^[2]^ Reported estimates of gout prevalence range from 2.7% to 6.7% in countries with a Western lifestyle. The most recent estimate (in 2015–2016) of the lifetime prevalence of gout in adults diagnosed by a health professional in the USA (3.9%) equates to 9.2 million individuals.^[3]^ This prevalence increases with age to 9% of adults >60 years of age in the USA. The most recent estimate of gout prevalence in mainland China is 1.1%.^[4]^

Currently, the main treatments for AGA include anti-inflammatory drugs (colchicine, non-steroidal anti-inflammatory drugs, and glucocorticoids) and urate-lowering drugs treatment (allopurinol, benbromarone, and febuxostat).^[5]^ However, the clinical application of these drugs are limited because of its adverse reactions such as severe gastrointestinal reactions and hepatic injury.^[6,7]^ Some studies have also reported serious adverse reactions on febuxostat and allopurino.^[8]^ Therefore, for acute gouty arthritis, it is necessary to seek a more effective treatment.

In recent years, the advantages of traditional Chinese medicine in preventing and treating such chronic diseases have been gradually recognized worldwide. In Chinese medicine, gouty arthritis is correlated with dampness, heat, sputum, and stasis. Among numerous effective prescriptions, Simiao pill, derived from Ermiao powder, and described in a famous traditional Chinese medicine monograph Chengfang Biandu in Qing Dynasty of China, was wildly applied for treatment of gouty arthritis.^[9]^ It is composed of 4 individual herbs: Rhizoma Atractylodis, Cortex Phellodendri, Radix Achyranthis Bidentatae, and Semen Coicis.

## 2 Methods

### 2.1 Protocol registration

The systematic review protocol has been registered on the INPLASY website as INPLASY202040163 (https://inplasy.com/inplasy-2020-4-0163/). Strictly follow the guidelines of Cochrane Handbook for Systematic Reviews of Interventions and the Preferred Reporting Items to conduct this Systematic Reviews and Meta-analysis Protocol (PRISM-P),^[10]^ and record important program revisions in the complete evaluation.

### 2.2 Inclusion criteria

#### 2.2.1 Study design

The study selected only clinical randomized controlled trials of si-miao-san decoction (SMSD) against AGA published in Chinese and English. Animal mechanism studies, reviews, case reports, and non-randomized clinical trials will be excluded.

#### 2.2.2 Participants

All adult patients (18 years and older, no upper age limit) with a diagnosis of acute gouty arthritis will be considered for this review. We will use the diagnostic criteria of the American College of Rheumatology (ACR) for AGA.^[11]^

#### 2.2.3 Interventions

Both groups received conventional gout treatment recommended by ACR guidelines, including diet and lifestyle.^[11]^ The experiment group used SMSD or modified SMSD, while the control group applied for no intervention, placebo, or conventional medication such as non-steroid anti-inflammatory drugs, colchicines, steroids, and adrenocorticotrophic hormone. In addition, the 2 groups did not take any drugs that interfered with the outcome indicators. The follow-up time was ≥4 weeks.

#### 2.2.4 Outcomes

The primary outcomes include the improvement in clinical efficacy, including pain score, joint function, and degree of swelling. The clinical efficacy refers to the guiding principles for clinical research of new Chinese medicines^[12]^ and is determined according to the degree of improvement of the symptoms of the patient before and after treatment: markedly effective: the clinical symptoms and signs of TCM improved significantly >70%; effective: the clinical symptoms and signs of TCM reduced by 30% to 70%; ineffective: the clinical symptoms and signs of TCM improved <30% or no improvement, or even worse. The nerve conduction velocity includes the sensory nerve conduction velocity and the motor nerve conduction velocity, which are evaluated by electromyography.

Secondary outcomes are mainly composed of blood uric acid (BUA), C-reactive protein (CRP), and erythrocyte sedimentation rate (ESR).

### 2.3 Search methods

#### 2.3.1 Electronic searches

We will retrieve each database from the built-in database until October 2020. Chinese literature comes from CNKI, Wanfang, VIP, and CBM databases. English literature mainly searches Cochrane Library, PubMed, Web of Science, and EMBASE. Different search strategies were combined as follows. For the English databases, we used free text terms, such as “simiao” “simiao san” “simiao san Decoction,” and “gouty arthritis” or “gout.” For the Chinese databases, free text terms were applied, such as “simiao” and “Tong Feng” (which means gout in Chinese). A filter for clinical studies was applied. We will simply present the search process of the PubMed library (Table 1). Adjusting different search methods according to different Chinese and English databases.

**Table 1:**
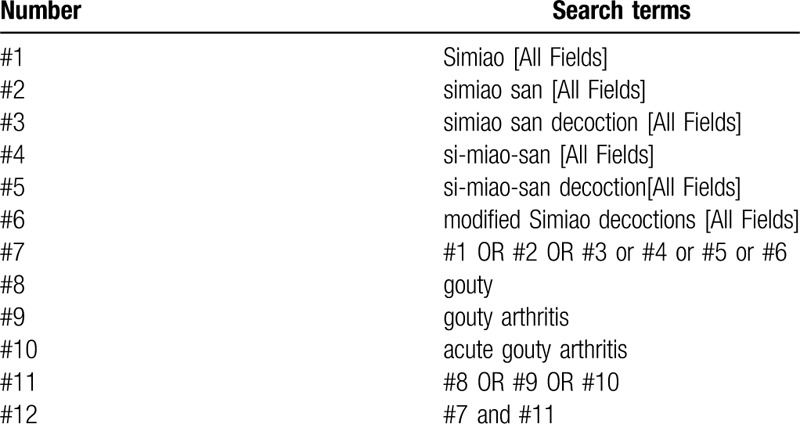
Example of PubMed search strategy.

#### 2.3.2 Searching other resources

At the same time, we will retrieve other resources to complete the deficiencies of the electronic databases, mainly searching for the clinical trial registries and grey literature about SMSD for AGA on the corresponding website.

### 2.4 Data collection and analysis

#### 2.4.1 Studies selection

**Figure 1:**
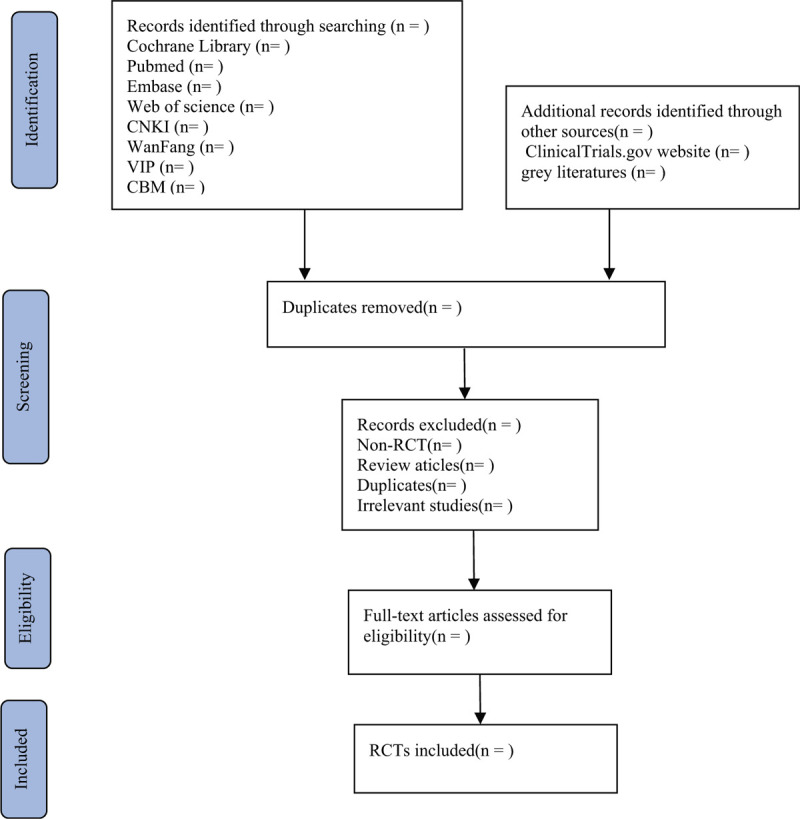
Flow chart of the study selection.

Two reviewers will independently retrieve all the literature. The references identifified from relevant database searches will be imported into the EndNote X9 software (Captivate Analytics, USA, version EndNote X9.3.2). The 2 reviewers will independently read the titles and abstracts of all references, and remove duplicate documents to determine the inclusion of the research-compliant literature. If there are any disagreements, the 2 researchers will discuss and reach an agreement. If no consensus can be reached, a third-party will be consulted to reach an agreement. A flow chart (Fig. 1) will be used to describe the identification and selection process of the study.

#### 2.4.2 Data extraction and management

According to the eligibility criteria, 2 reviewers will evaluate the studies by using the same eligibility evaluation form. The following information will be documented from all the included studies: study characteristics (title, first author, publication year, study design, sample size, setting, randomization methodology, allocation concealment, blinding), participant characteristics (age, sex, number in each group, etc), intervention details (type of interventions, type of controls, dose, route of administration, duration of treatment or follow-up, number of cases included in the statistical analysis, etc), outcome indicators for efficacy and safety. Any disagreements will be rechecked and discussed. If no agreement can be achieved, the final decision will be consulted with a third reviewer.

#### 2.4.3 Assessment of risk of bias

All the included studies will be evaluated based on the guidelines of Cochrane Handbook for Systematic Reviews of Interventions.^[13]^ The quality of each trial will categorized into “low,” “unclear,” or “high” risk of bias according to the following items: adequacy of generation of the allocation sequence, allocation concealment, blinding of participants and personal, blinding of outcome assessors, incomplete outcome data, selected reporting the results, and other sources of bias (such as comparable baseline characteristic, inclusion, and exclusion criteria).

#### 2.4.4 Measures of treatment effect

Select the evaluation method according to different curative effect indexes. For the dichotomous data, we will choose the relative risk (RR) of the indicator of the effect scale with 95% confidence interval (CI). For continuous data expressed as mean difference (MD) or standard mean difference (SMD), the CI value is 95%, depending on whether the measurement range is consistent.

#### 2.4.5 Dealing with missing data

If the missing relevant data is still not available after contacting the author, we can synthesize the available data in the initial analysis. In addition, sensitivity analysis will be used to assess the potential impact of missing data on the overall results of the study.

#### 2.4.6 Data analysis

We use Review Manager software version 5.3 provided by Cochrane Collaboration to analyze the data. The RR of 95% confidence interval (CI) was used to summarize the dichotomous data. Continuous data will be summarized by using the weighted mean difference of 95% CI. According to research recommendations,^[14]^ we will use a random effects model for meta-analysis in this paper.

Statistical heterogeneity will be evaluated by Chi-square test and *I*^2^ test. *P*-value ≥.1 and *I*^2^ ≤ 50% indicate that the study has no significant statistical heterogeneity. In contrast, *P*-value <.1 or *I*^2^ > 50%, indicating that there is considerable heterogeneity. When there is no statistical heterogeneity, a fixed effect model (FEM) will be used. In contrast, when there is a statistical heterogeneity, a random effect model (REM) will be used. In addition, we will conduct subgroup or sensitivity analysis to find potential causes. If meta-analysis can not be performed, we will conduct a descriptive analysis.

#### 2.4.7 Subgroup analysis

We will conduct subgroup analysis based on different reasons such as age, sex, different forms of intervention, drug dosage form, dosage, treatment process, etc.

#### 2.4.8 Sensitivity analysis

To evaluate the robustness of the meta-analysis results, we will first delete the low-quality studies and then merge the data to assess the impact of the sample size, study quality, statistical methods, and missing data on the meta-analysis results.

#### 2.4.9 Reporting bias

If there are >10 studies in the meta-analysis, the symmetry of the funnel plot will be assessed to examine publication bias, with results being interpreted cautiously.

#### 2.4.10 Grading the quality of evidence

The investigator will use “the Grading of Recommendations Assessment, Development and Evaluation system (GRADE)” to independently assess the quality of evidence for each result.^[15]^ The GRADE system divides the quality of evidence into 4 levels: high, medium, low, and very low. GRADE profiler 3.2 will be used for analysis.

## 3 Discussion

Gout is a metabolic disease. The joint redness and dysfunction caused by the disease seriously affects the quality of life of patients. Although the current mainstream drugs have definite effects but obvious side effects, Clinicians need to find drugs with better efficacy and fewer side effects. Chinese herbal medicine Si-Miao-San Decoction has been used to Gouty arthritis for many years in China.^[16–19]^ At present, there is no evidence-based medicine to confirm the efficacy of SMSD on AGA. Therefore, we try to conduct this meta-analysis to provide high-quality evidence on the clinical efficacy and safety of SMSD, and hope to promote the application of traditional Chinese medicine and benefit more patients.

## Author contributions

**Conceptualization:** Heting Wang, Xiyu Zhang.

**Data curation:** Shiyin Chen.

**Formal analysis:** Yong Luo.

**Funding acquisition:** Xiyu Zhang.

**Methodology:** Yuan Zhang.

**Project administration:** Heting Wang, Hua Duan.

**Resources:** Qingsong Liu, Xiyu Zhang.

**Software:** Qingsong Liu, Xiyu Zhang.

**Supervision:** Shiyin Chen, Qingsong Liu.

**Writing – original draft:** Heting Wang, Hua Duan.

**Writing – review & editing:** Xiyu Zhang.
